# 

**Published:** 1915-10-30

**Authors:** 


### CONTENTS.

| | PAGE |
|---|---|
| **Heroism in Hospital Workers** ... ... ... | 91 |
| **Medical Service during War and Peace** ... | 92 |
| **Hospital and Institutional News** ... ... ... | 93 |
| His Majesty's Gift to the Canadian Red Cross—A Big Step Forward—Ophthalmic Surgery by Nurses!—King Edward VII.'s Hospital, Cardiff—Poor-Law Amalgamation in Glasgow—Rancid Butter at Bermondsey—The M.O.H.'s Sarcasms—Should the Birmingham Orthopædic Hospital be Rebuilt?—The Planning of Open-Air Hospitals—Red Cross Day in the Streets—Fifty Motor Ambulances Sunk—Suspension of School Medical Inspection—The Prospects of Another Spotted Fever Outbreak—Poor-Law Relief for Disabled Soldiers—The London Ambulance Service—The Red Cross at Burlington House—Death of a Hospital Commandant—The Forfarshire Local Authorities and Sanatorium Treatment—The Archbishop at the Croydon General Hospital—A Spontaneous and Inspiring Gift—The late Dr. W. G. Grace—Hospital Launches for the Dardanelles—Expansion at Toxteth Infirmary—Increased Prices at Leicester Infirmary.—The Pay of the *Locum Tenens*—The War and Public Health—The Wollaston War Hospital Opened—Damages Against an "Institute"—This Week's Drug Market. | |
| **Rheumatism in its Professional and Popular Aspects** ... ... ... ... ... ... ... | 99 |
| **A Medical Causerie** ... ... ... ... ... | 101 |
| **Parliament and the Profession** ... ... ... | 102 |
| **The War and Voluntary Hospital Beds** ... | 103 |
| Military Convalescents *v.* Urgent Cases. | |
| **The Grievances of Poor-Law Medical Officers** | 104 |
| **National Insurance Act Developments** ... ... | 105 |
| Some Problems Which Press. | |
| **Cubic Air Space for Institutional Infants** ... | 106 |
| **Under the Red Cross** ... ... ... ... ... | 107 |
| A Record of Wonderful Achievements. | |
| **The Editor's Letter-Box** ... ... ... ... | 108 |
| **Institutional Needs** ... ... ... ... ... ... | 108 |
| **Patriotism and the Nurse-Training Schools** ... | 109 |
| II.—The London Hospital. | |
| **Passing Events and Topics** ... ... ... ... | 110 |
| **The Institutional Worker** ... ... (Suppl.) | 1 |
| Work for Consumptives in Sanatoria:—Answer to September Question. | |
| Question for October ... ... ... (Suppl.) | 2 |
| Questions Invited ... .. ... (Suppl.) | 2 |
| Enquiries and Answers ... ... ... (Suppl.) | 2 |
| The Housekeeper's Department. | |
| The Sick and In Need. | |
| War Matters. | |
| Miscellaneous | |
| Practical Matters. | |

